# Optic neuropathy associated with GVHD after bone marrow allogeneic hematopoietic stem cell transplantation for B-Acute lymphoblastic leukemia: case report

**DOI:** 10.1186/s12886-022-02283-4

**Published:** 2022-03-01

**Authors:** Bowen Wang, Fagang Jiang, Xinghua Wang

**Affiliations:** Department of Ophthalmology, Union Hospital, Tongji Medical College, Huazhong University of Science and Technology, Wuhan, 430022 China

**Keywords:** Optic nerve, Graft-versus-host disease, Bone marrow allogeneic hematopoietic stem cell transplantation, Case report

## Abstract

**Background:**

Graft-versus-host disease (GVHD) is the most common complication after bone marrow allogeneic hematopoietic stem cell transplantation (allo-HSCT). The incidence of posterior segment complications was significantly lower than that of ocular surface lesions. Up to now, there has been no report about optic neuropathy associated with GVHD.

**Case presentation:**

A 23-year-old man presented with visual acuity decline after allo-HSCT for B-acute lymphoblastic leukemia (B-ALL). Red rashes were found all over the body simultaneously. Visual field examination revealed central scotomas in both eyes. Visual evoked potential showed prolonged P100 latency and decreased P100 amplitude in both eyes. Other ocular examinations showed no obvious abnormality except for blunt pupillary light reflex. The minimal residual disease test was negative after transplantation, and no obvious abnormalities were found in optic nerve and brain by magnetic resonance imaging (MRI). After the multi-disciplinary consultation, the rashes and optic neuropathy were considered GVHD probably. As for the treatment, methylprednisolone and Ruxolitinib were suggested, supported by adjunctive neurotrophic therapy. Two months later, the rashes gradually subsided. However, the visual acuity was not significantly improved at latest follow-up.

**Conclusions:**

The present case report demonstrated GVHD probably associated with optic neuropathy. Although extremely rare, optic nerve should be considered as a potential target of ocular GVHD, which could expand the dimensions of GVHD.

## Background

Acute lymphoblastic leukemia (ALL) represents 12% of all leukemia cases, with a worldwide incidence of 1–4.75 per 100,000 people [1]. Bone marrow allogeneic hematopoietic stem cell transplantation (allo-HSCT) is the main strategy of ALL treatment except for classic chemotherapy. Graft-versus-host disease (GVHD) is the most common complication after allo-HSCT. The incidence of ocular GVHD (oGVHD) after bone marrow transplantation is as high as 60–90% [2], which could involve cornea, conjunctiva, uvea, retina, lacrimal gland and meibomian gland. The incidence of posterior segment complications including uveitis, giant cell retinitis and retinal hemorrhage was significantly lower than that of ocular surface lesions, such as dry eye and meibomian gland dysfunction (MGD) [3]. So far, there have been no report of GVHD involving the optic nerve. Here, we reported a case of optic neuropathy probably associated with GVHD after allo-HSCT for B-ALL treatment.

## Case presentation

**Fig. 1:**
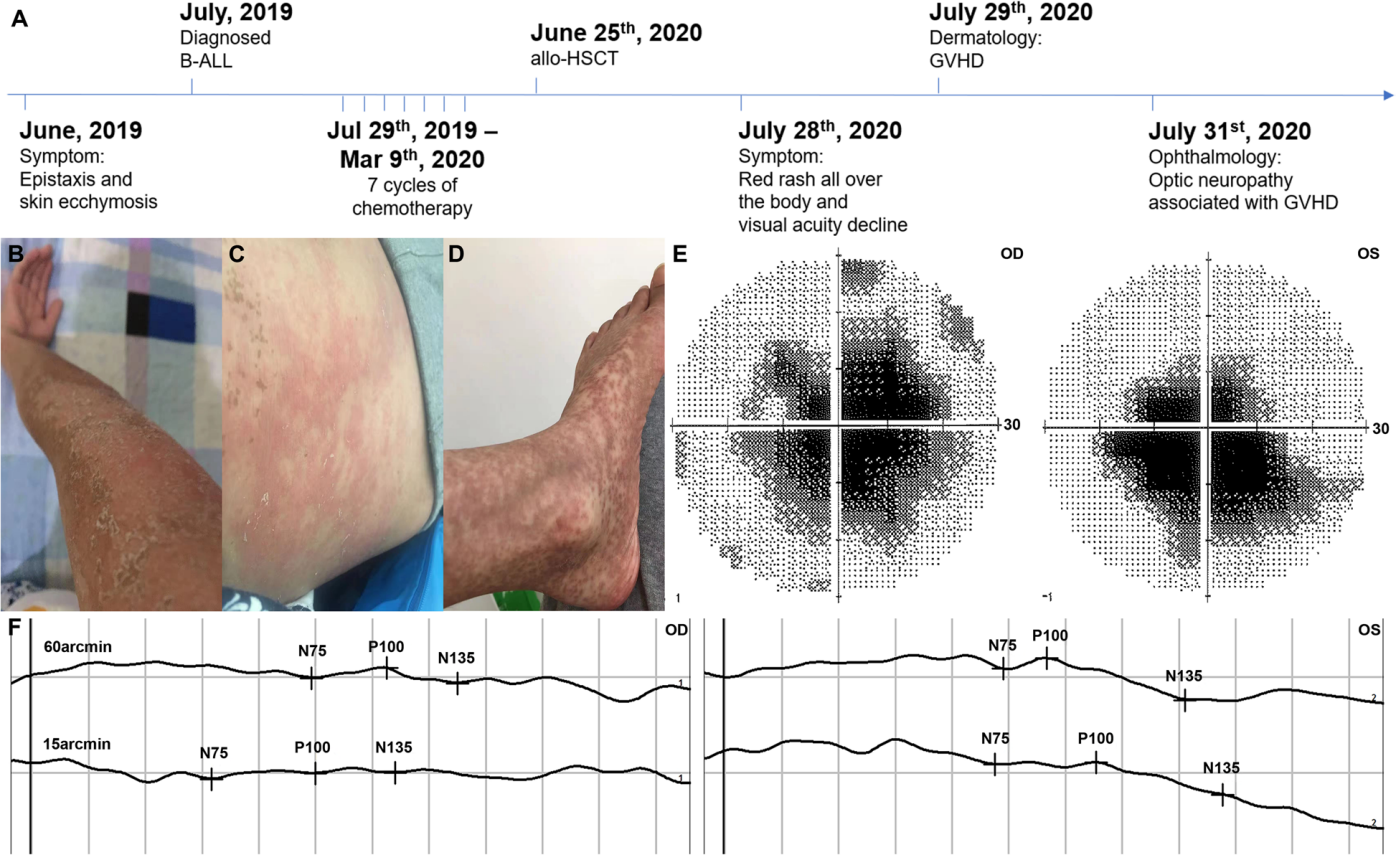
Optic neuropathy and skin disease associated with GVHD. **A** The disease process of the patient. **B**, **C**, **D** Scattered red rashes all over the body, including right arm **B**, waist **C**, left lower leg and left foot (**D**). Visual field (**E**) revealed central scotomas. VEP **F** showed prolonged P100 latency and decreased P100 amplitude at 60 and 15 arcmin. Abbreviations: GVHD, graft-versus-host disease; VEP, visual evoked potential; OD, oculus dextrus; OS, oculus sinister

A 23-year-old man was admitted to Department of Ophthalmology for visual acuity decline in both eyes on July 28^th^, 2020, without other visual disturbances, headache or the other symptoms. He received allo-HSCT for ALL treatment one month ago. He reported that he was previously healthy, and his uncorrected visual acuity was 20/20 before. As for the past history of ALL (Fig. 1A), the patient was diagnosed with B-ALL because of "epistaxis and skin ecchymosis" in June, 2019. Subsequently, 7 cycles of hyper-CVAD chemotherapy were performed (from Jul 29^th^, 2019 to Mar 9^th^, 2020), followed by bone marrow transplantation on June 25^th^, 2020. One month later, red rashes were found all over the body, protruding from the surface of the skin (Fig. 1B, C, D).

**Fig. 2:**
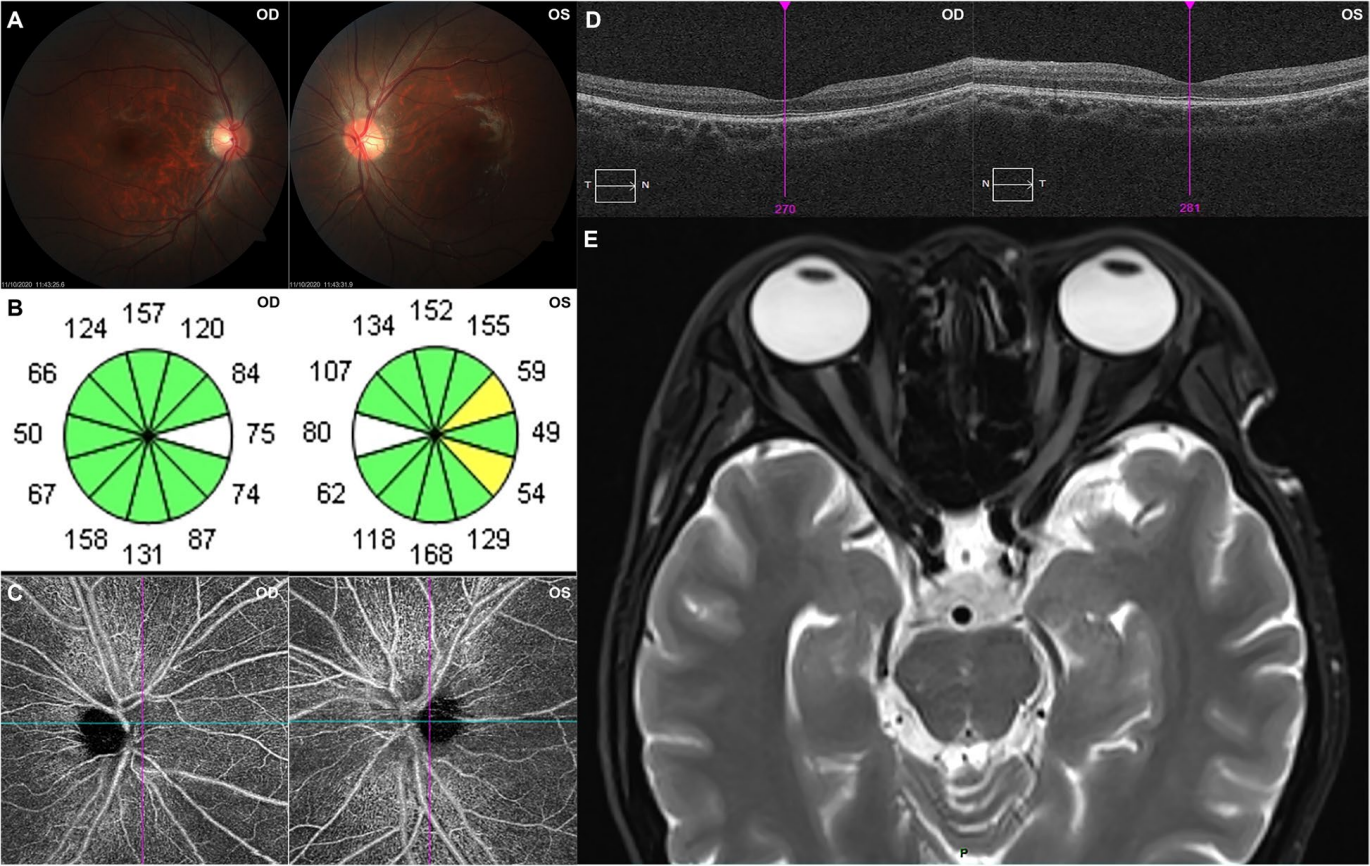
Ocular examinations and MRI. Fundus photography **A** showed optic disc with normal morphology. OCT **B** showed normal RNFL thickness. OCTA **C** showed normal capillary perfusion density. Macular OCT **D** showed normal morphology. MRI **E** showed no noticeable abnormality in optic nerve and brain. Abbreviations: OCT, optical coherence tomography; RNFL, retinal nerve fiber layer; OCTA, optical coherence tomography angiography; MRI, magnetic resonance imaging; OD, oculus dextrus; OS, oculus sinister

Immediately, the ocular examinations were performed. The visual acuity was 20/300 without improvement by correction in both eyes. The intraocular pressure is 15 mmHg in right eye and 16 mmHg in left eye. The anterior segment was normal in both eyes, except for blunt pupillary light reflex (both direct and indirect). Fundus photography showed normal optic disc morphology with clear boundary, without obvious pallor (Fig. 2A). Visual field examination revealed central scotomas in both eyes (Fig. 1E). Visual evoked potential (VEP) showed prolonged P100 latency and decreased P100 amplitude at 60 and 15 arcmin in both eyes (Fig. 1F). The average retinal nerve fiber layer (RNFL) thickness was 99 μm and 106 μm in right and left eye, respectively (Fig. 2B). Macular optical coherence tomography (OCT) showed normal morphology (Fig. 2D). Optic disc OCT Angiography (OCTA) also showed normal capillary perfusion density (Fig. 2C). Meanwhile, there was not noticeable abnormality in optic nerve and brain by MRI (Fig. 2E). A cerebrospinal fluid (CSF) examination revealed no significant abnormality. The AQP4, MOG, GFAP and MBP tests were negative in both blood and CSF. Beside this, bacteria and fungi blood culture, as well as IgG and IgM of several viruses were performed to exclude the infectious etiology, which showed negative results.

After the multi-disciplinary consultation combined with departments of hematology, dermatology and ophthalmology, the rashes and optic neuropathy were considered GVHD probably, despite the routine use of anti-rejection medications after allo-HSCT, such as Cyclosporin A (CsA). As for the treatment, systematic steroid and neurotrophic medication were suggested, accompanied by potassium, calcium supplement medication and gastrointestinal protection medication until steroid withdrawal. In addition, Ruxolitinib was recommended by department of dermatology. Systematic steroid therapy lasted for one month from Aug 1^st^, 2020, while neurotrophic medication and Ruxolitinib continued with routine anti-rejection medication under regular follow-ups. We rechecked the ocular and dermal situations every week during the first month follow-ups, followed by every month in the next six months, and then every three months regularly. Two months later, the rashes gradually subsided. However, the visual acuity was not significantly improved until his last follow-up at Aug 10^th^, 2021.

## Discussion and conclusion

Optic neuropathy is a refractory ocular disease, which could cause visual acuity decline, visual field defect, and even blindness. Optic neuropathy could be induced by many etiologies, including heredity, inflammation, infection, ischemia, trauma, medication toxicity, and mechanical compression caused by orbital or intracranial space-occupying lesion [4].

In this case, the patient presented with simultaneous optic neuropathy and dermal red rashes. As for the cause of his optic neuropathy, we could exclude hereditary and traumatic etiology by the negative past medical history of this young man. Further, we exclude mechanical compression of optic nerve by the negative results of MRI. Subsequently, negative results of common pathogens (including bacteria, virus and fungi) indicated that infection could be excluded. By fundus photography, OCT, OCTA and ultrasound images, ischemia optic neuropathy could be excluded. Besides this, we performed AQP4, MOG, GFAP and MBP tests in both blood and CSF samples to exclude neuromyelitis optica spectrum disorder, which showed negative results.

As for the optic neuropathy associated with ALL and related treatments, the most common mechanism is the direct involvement of the optic nerve by ALL [5]. In this case, the patient underwent several times of bone marrow smears for ALL monitoring. The minimal residual disease test was negative after transplantation, and no obvious abnormalities were found in optic nerve and brain by MRI, which could exclude ALL relapse.

Until now, no reference was found which reported optic neuropathy caused by hyper-CVAD or allo-HSCT, except Sbeity ZH [6] reported a woman with long-standing Methotrexate (MTX) suffered from an acute central visual field defect. MTX-induced optic neuropathy was suspected, and the patient experienced improvement only six weeks after discounting MTX therapy. In this case, patient suffered from visual acuity decline 4 months after chemotherapy, which indicated less possibility of chemotherapy causing optic neuropathy.

As for the potential neurotoxicity of CsA, Mimouni K [7] reported that optic neuropathy was significantly associated with older age, and it has been reported that CsA might induce optic neuropathy through possible mechanism of microangiopathy [8]. However, the patient in this case adjusted the dosage of CsA by regularly monitoring the plasma concentration of CsA, keeping it normal, which indicated less possibility of CsA neurotoxicity combined with the normal OCTA results. Interestingly, the patient continued CsA medication, the red rashes subsided, but the optic neuropathy did not get better or worser, which reminded us that optic neuropathy was unlikely caused by CsA.

Moreover, the visual acuity decline and red rashes presented simultaneously after bone marrow transplantation. The chimerism rate of the donor in this case was 97.3%, suggesting that the bone marrow was not fully implanted. Based on these evidences, we concluded that GVHD probably involved both optic neuropathy and dermal disorder.

GVHD is the most common complication after allo-HSCT, which is mediated by immune disorders and tissue inflammation involved in single or multiple systems, leading to tissue fibrosis and organ dysfunction [9]. After HSCT, the incidence of GVHD is as high as 40%-60% [3]. GVHD that occurs within 100 days of HSCT is called acute GVHD (aGVHD). Clinical manifestations of systemic aGVHD mostly involve skin, gastrointestinal tract, and liver. Although rare, GVHD could also attack the nervous system, including peripheral or central nervous system. Kraus PD [10] reported 27 GVHD patients who complained of symptoms indicating peripheral nervous system complications. Suzuki S [11] reported a woman who presented polyneuropathies after HSCT. Diffuse erythema, diarrhea, muscle numbness, weakness and quadriplegia appeared successively, and she was diagnosed as “chronic GVHD and inflammatory demyelinating polyneuropathy”.

As for the ocular GVHD (oGVHD), acute oGVHD is a relatively rare manifestation of aGVHD with an incidence of about 7.2% among allo-HSCT patients [5, 8]. The incidence of chronic oGVHD has been reported to be about 50–80%, which is higher than acute oGVHD [12]. Ocular GVHD could involve cornea, conjunctiva, uvea, retina, lacrimal gland and meibomian gland. The incidence of posterior segment complications including uveitis, giant cell retinitis and retinal hemorrhage was significantly lower than that of ocular surface lesions, such as dry eye and meibomian gland dysfunction (MGD) [2]. The diagnosis of oGVHD is mainly based on the relevant ocular symptoms, signs and biopsies. oGVHD should be differentiated from ocular infiltration of leukemia, which may occur in 9–90% of cases, and can involve orbit, uveal tract, retina and optic nerve [13, 14]. In addition to ocular symptoms, there could be definite abnormalities in hemogram, bone marrow and CSF. It has been reported that cytokines such as IL-2, IL-6 and IL-8/CXCL-8 in tears are expected to be biomarkers of oGVHD [15]. In addition, neutrophil elastase (NE), myeloperoxidase (MPO) and matrix metalloproteinase (MMPs) were found to be significantly increased in tears from GVHD patients [16]. The treatment of oGHVD includes local and systemic treatment, including immunosuppressive and anti-rejection treatment. We suggest that patients with oGVHD should be treated with multidisciplinary collaborative therapy and personalized treatment to improve the prognosis of patients.

Although it hasn’t been reported, the possibility of GVHD attacking optic nerve could be theoretical, similar to GVHD attacking peripheral or central nervous system. To the best of our knowledge, this is the first report of GVHD probably involving the optic nerve, which could expand the dimensions of oGVHD. The present case indicated that GVHD involving optic neuropathy could be accompanied with other tissue and organ disorders caused by GVHD. As for the mechanism involving GVHD-induced optic neuropathy, inflammation should be considered because of the immune system hyperactivity in GVHD patients. We suggest regular ophthalmic examinations should be taken in leukemia patients, to detect subclinical ocular involvement or relapse during complete remission of leukemia.

## Data Availability

The authors declare that all data supporting the findings of this study are available within the article.

## Abbreviations

GVHD: Graft-versus-host disease
allo-HSCT: Allogeneic hematopoietic stem cell transplantation
B-ALL: B-acute lymphoblastic leukemia
MRI: Magnetic resonance imaging
ALL: Acute lymphoblastic leukemia
oGVHD: Ocular GVHD
MGD: Meibomian gland dysfunction
VEP: Visual evoked potential
RNFL: Retinal nerve fiber layer
OCT: Optical coherence tomography
OCTA: OCT Angiography
CSF: Cerebrospinal fluid
OD: Oculus dextrus
OS: Oculus sinister
CsA: Cyclosporin A
aGVHD: Acute GVHD
NE: Neutrophil elastase
MPO: Myeloperoxidase
MMPs: Matrix metalloproteinase
